# Résultats de la prise en charge médico-chirurgicale en urgence des lésions œsophagiennes liées aux ingestions de produits caustiques chez l'enfant dans le service des urgences de l'Hôpital général de référence de Niamey (Niger)

**DOI:** 10.48327/mtsi.v4i1.2024.399

**Published:** 2024-02-01

**Authors:** Rabiou SANI, Aliou ZABEIROU, Illé SALHA, Ibrahim ISS OUF OU ALZOUMA, Boubé DJAFAROU ABARCHI, Lassey JAMES DIDIER, Rachid SANI, Habibou ABARCHI

**Affiliations:** 1 Service de chirurgie générale et digestive, Hôpital général de référence, Niamey, Niger; 2 Service de chirurgie thoracique et cardiovasculaire, Hôpital général de référence, Niamey, Niger; 3 Service dotorhinolaryngologie, Hôpital général de référence, Niamey, Niger; 4 Faculté des sciences de la santé, Université Abdou Moumouni, Niamey, Niger

**Keywords:** Œsophagite, Ingestion caustique, Sténose œsophagienne, Fibroscopie, Œsophagoplastie, Hôpital, Niamey, Niger, Afrique subsaharienne, Esophagitis, Caustic ingestion, Oesophageal stricture, Endoscopy, Esophagoplasty, Hospital, Niamey, Niger, Sub-Saharan Africa

## Abstract

**Introduction:**

L'ingestion de produits caustiques chez l'enfant constitue un problème de santé publique; il s'agit essentiellement d'accidents domestiques dus aux mauvais conditionnements et aux stockages de ces produits. C'est une urgence médico-chirurgicale dont la prise en charge est multidisciplinaire. Les lésions occasionnées peuvent engager le pronostic fonctionnel œsophagien et vital dans 10 % des cas.

**Méthodologie:**

Il s'agissait d'une étude rétrospective et analytique, sur une période de 2 ans (janvier 2020 à décembre 2022), réalisée dans le service des urgences de l'Hôpital général de référence de Niamey (Niger). Cette étude avait inclus les patients de moins de 15 ans admis pour ingestion de produit caustique.

**Résultats:**

Notre étude avait inclus 17 patients; l’âge moyen était de 5 ans (2-11 ans); le sexratio (H/F) était de 2,4. La soude caustique était en cause dans 59 % des cas, et le délai moyen de consultation était de 3 jours. La dysphagie était présente dans 76 % des cas. L’état de choc et la dénutrition sévère étaient retrouvés dans respectivement 18 et 24 % des cas. À l'examen abdominal, 12 % des patients présentaient une défense abdominale. L'endoscopie était réalisée chez 4 patients et les lésions sévères œsophagiennes (IIIb de Zargar) étaient retrouvées dans 25 % des cas. Sur le plan thérapeutique, l'antibioprophylaxie et la corticothérapie avaient été réalisées dans respectivement 71 % et 53 % des cas. Les dilatations endoscopiques avaient été réalisées dans 12 % des cas et la chirurgie était indiquée chez 7 patients (41 %) dont 3 gastrostomies d'alimentation transitoires, 3 œsophagoplasties par transplant colique et 1 stripping de l’œsophage.

**Conclusion:**

Les ingestions accidentelles caustiques peuvent avoir des conséquences graves. La prévention de ces accidents repose sur la sensibilisation du public sur les dangers associés au stockage inapproprié de ces produits.

## Abstract (en)

**Introduction:**

Caustic ingestion in children is a public health problem; it is mainly due to domestic accidents due to improper packaging and storage of caustic products. It is a medical and surgical emergency whose management is multidisciplinary. The lesions caused by the accidental ingestion of caustics can affect the functional and vital prognosis in 10% of cases.

**Methodology:**

A retrospective, descriptive study from January 2020 to December 2022 (2 years), carried out in the emergency department of the General Reference Hospital of Niamey (Niger). The study included patients less than 15 years old admitted for ingesting a caustic product.

**Results:**

Our study included 17 patients. The average age was 5 years, with age extremes of 2 to 11 years. We noted a male predominance with a sex ratio (M/F) of 2.4. Ingestion of caustic products was accidental in all cases. The caustic product was caustic soda in 59%. The average quantity of product ingested was 5 ml (2 ml to 20 ml). The average consultation time was 3 days (3 hours to 15 days). Clinically, dysphagia was the most functional sign, represented by 13 cases, or 76%. Regarding general signs, 3 patients (18%) were admitted with fever; blood pressure was normal in 15 patients (88%); and 2 patients (18%) were admitted in a state of shock. The respiratory rate was normal in 14 patients (82%). Four patients (24%) were admitted in a state of deterioration in the general condition associated with severe malnutrition and dehydration. On physical examination, 2 patients (12%) presented with abdominal defense at the epigastric level. Examination of the ENT sphere revealed benign buccopharyngeal ulcerations in 2 patients (12%). Esogastroduodenal fibroscopy was performed in 4 patients (24%). The caustic lesions observed in the esophagus were: Zargar stage I at 25%, stage Ila at 50%, and stage Illb at 25%. In the stomach, the lesions were Zargar stage I in 75% of cases and stage III in 25% of cases. An injected thoracic-abdominopelvic computed tomography (CT) was performed in 3 patients (18%). It revealed a lack of enhancement of the esophageal wall compatible with esophageal necrosis in one patient. An esophagogastroduodenal transit was performed in 8 patients (47%) admitted more than 72 hours after ingestion of the caustic. They showed esophageal stenoses longer than 3 cm in 3 patients, multiple esophageal stenoses in 2 patients, a single esophageal stenosis in 2 patients, and a single antropyloric stenosis in 1 patient. Therapeutically, all patients benefited from antiemetics to avoid vomiting and proton pump inhibitors. Intravenous antibiotic prophylaxis with third-generation cephalosporin was administered to 12 patients (71%). Corticosteroid therapy based on IV prednisolone at a dose of 1 g/1.73 m^2^ per day was used to limit or prevent stenoses in 9 patients (53%). Parenteral nutrition was administered to 7 patients (41%). Endoscopic dilations were performed in 2 patients (12%). Emergency surgical treatment was performed in 7 patients (41%): 3 patients underwent transitional feeding gastrostomies; in 3 others, esophagoplasties by colon transplant were performed, and 1 patient was treated by stripping of the esophagus associated with total gastrectomy. The postoperative course was marked by a leak of esocolic anastomosis in one patient for whom conservative treatment was performed with good progress. The average length of hospital stay was 5 days (1-32 days).

**Conclusion:**

Accidental caustic ingestions can have serious consequences. Preventing these accidents relies on raising public awareness of the dangers associated with improper storage of these products.

## Introduction

Les lésions caustiques de l’œsophage sont définies comme l'ensemble des lésions tissulaires intéressant l’œsophage en rapport avec une agression chimique, dues à l'ingestion volontaire ou accidentelle d'un produit caustique [5].

Sur le plan épidémiologique, la mortalité globale par ingestion caustique à l’échelle mondiale était estimée par l'Organisation mondiale de la santé en 2004 à 310 000 personnes, soit un taux de mortalité de 4,8/100 000 par an, dont 30 % étaient des enfants. Les séquelles à long terme, dominées par les sténoses œsophagiennes, sont estimées à 73 % [10]. Chez l'enfant, l'ingestion de caustique est généralement due à des accidents domestiques. Ces accidents deviennent de plus en plus fréquents du fait de l’évolution du mode de vie (conditionnement inadapté, stockage à des endroits accessibles aux petits enfants, vente libre des produits caustiques…). Les ingestions volontaires de produits caustiques, dans un but d'autolyse (suicide), sont l’œuvre des adultes [18]. Le délai écoulé entre l'ingestion du caustique et la prise en charge initiale constitue un facteur pronostic important. La prise en charge est multidisciplinaire, impliquant médecins urgentistes, gastro-entérologues, réanimateurs, chirurgiens, oto-rhino-laryngologistes et psychiatres [13]. Cette étude a pour but d'analyser les résultats de la prise en charge médico-chirurgicale de la phase aiguë des lésions œsophagiennes liées aux ingestions de produits caustiques chez l'enfant.

## Méthodologie

Nous avons mené une étude rétrospective et analytique au sein du service des urgences de l'Hôpital général de référence de Niamey (Niger) sur une période de 2 ans (janvier 2020 à décembre 2022), incluant les patients de moins de 15 ans admis aux urgences pour ingestion de produit caustique. Les données des patients ont été collectées à partir des dossiers d'hospitalisation des patients, des comptes rendus opératoires et des fiches d'anesthésie. Une fiche d'enquête préétablie avait servi pour la collecte des données. Au total, 17 patients avaient été inclus. Nous avons utilisé la classification endoscopique et tomodensitométrique de Zargar (Tableau I) [20] et l'algorithme de prise en charge de Chirica [3].

**Tableau I:** Classifications endoscopique et tomodensitométrique selon Zargar Endoscopic and computed tomography classifications according to Zargar

**Classification endoscopique des lésions caustiques selon Zargar**	
Stade 0 : normal	
Stade I : œdème et érythème	
Stade II	a. hémorragies muqueuse, ulcérations superficielles, fausses membranes
	b. ulcérations creusantes et confluentes	
Stade III	a. nécrose focale non circonférentielle
	b. nécrose diffuse circonférentielle	
**Classification des lésions caustiques sur le scanner selon Zargar**	
Stade 1 : organes normaux, correspond aux stades endoscopiques 0-IIa de Zargar	
Stade 2 : œdème de la paroi avec altération inflammatoire des organes dans lentourage avec prise de contraste augmentée, correspond au stade endoscopique IIb-IIb, sans nécrose transmurale	
Stade 3 : nécrose transmurale, pas de prise de contraste, correspond au stade IIIb	

La prise en charge était assurée par les gastroentérologues, les chirurgiens, les réanimateurs et les ORL. Nous avons analysé les données épidémiologiques, cliniques, paracliniques, thérapeutiques et évolutives. Ces données ont été saisies dans un tableau Excel puis analysées par le logiciel SPSS.

## Résultats

**Figure 1:**
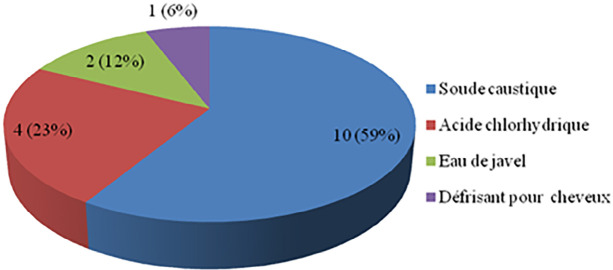
Répartition des patients selon le produit caustique ingéré Distribution of patients according to the type of caustic product ingested

**Figure 2:**
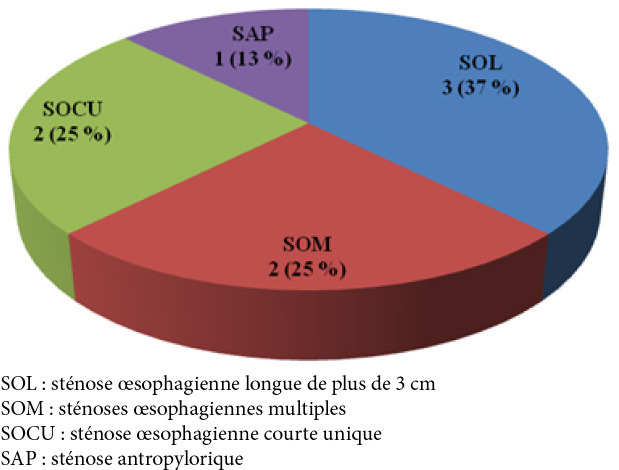
Répartition des patients selon le résultat du transit œso-gastro-duodénal (TOGD) Distribution of patients according to the result of the esophagogastroduodenal transit

**Figure 3:**
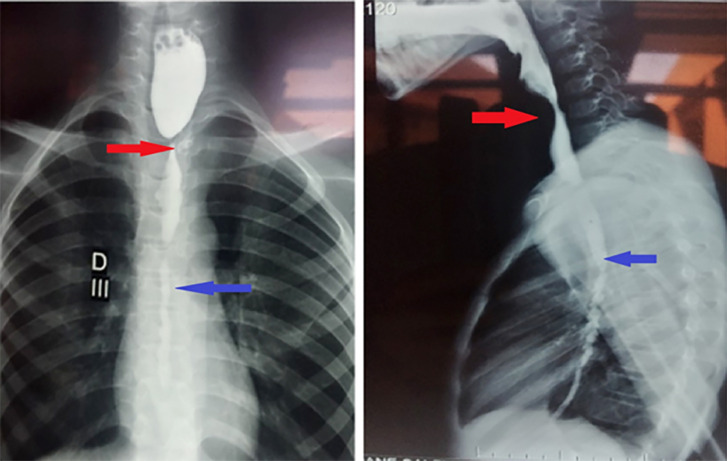
Cliché de TOGD face et profil, montrant une sténose de l’œsophage cervical (flèches rouges), associée à un long rétrécissement de l’œsophage thoracique (flèches bleues) Face and profile esophagogastroduodenal transit radiograph, showing stenosis of the cervical oesophagus (red arrows), associated with a long narrowing of the thoracic oesophagus (blue arrows)

Notre étude a inclus 17 patients. La moyenne d’âge était de 5 ans avec des extrêmes de 2 à 11 ans. Nous avons noté une prédominance masculine avec un sex-ratio (H/F) de 2,4. L'ingestion de produit caustique était accidentelle dans tous les cas. Le produit caustique le plus fréquemment en cause était la soude caustique dans 59 % des cas (Fig. 1). La quantité moyenne de produit ingérée était de 5 ml (2 ml à 20 ml). Le délai de consultation moyen était de 3 jours (3 h à 15 jours) avec une médiane de 2 jours (les interquartiles 25-75 % de 3 jours). Sur le plan clinique, la dysphagie était le signe fonctionnel le plus représenté avec 13 cas, soit 76 % (Tableau II). En ce qui concernait les signes généraux, 3 patients (18 %) ont présenté une fièvre à leur admission et 4 patients (24 %) ont été admis dans un état de dénutrition sévère et de déshydratation (Tableau III). À l'examen physique, 2 patients (12 %) présentaient une défense au niveau épigastrique. L'examen de la sphère ORL a révélé des ulcérations bucco-pharyngées bénignes chez 2 patients (12 %). La fibroscopie œso-gastro-duodénale a été réalisée chez 4 patients et a révélé des lésions œsophagiennes de stade IIIb dans 25 % (Tableau IV). Une radiographie du thorax centrée sur les coupoles diaphragmatiques a été pratiquée systématiquement sans montrer d'anomalie chez aucun patient. Une tomodensitométrie (TDM) thoraco-abdomino-pelvienne injectée a été réalisée chez 3 patients (18 %). Elle a mis en évidence un défaut de rehaussement de la paroi œsophagienne compatible avec une nécrose œsophagienne chez un patient. Un transit œso-gastro-duodénal (TOGD) a été réalisé (Fig. 2) chez 8 patients (47 %) admis plus de 72 heures après l'ingestion du caustique. Il a montré des sténoses œsophagiennes longues de plus de 3 cm chez 3 patients (Fig. 3).

**Tableau II:** Répartition des patients selon les signes fonctionnels retrouvés Distribution of patients according to functional signs

Signe fonctionnel	Effectif	Pourcentage (%)
Dysphagie	13	76
Vomissement	12	71
Hyper-sialorrhée	12	71
Douleur abdominale	4	24
Douleur rétrosternale	2	12
Hématémèse	1	6
Fausses routes	1	6

**Tableau III:** Répartition des patients selon les signes généraux retrouvés à l'admission Distribution of patients according to the general signs found on admission

Signe général	Effectif	Pourcentage (%)
Fièvre	3	18
Hypotension	2	12
Dyspnée	3	18
État de choc	3	18
Dénutrition sévère	4	24
Déshydratation	4	24
Trouble de conscience	1	6

**Tableau IV:** Répartition des patients selon les résultats de l'endoscopie (classification de Zargar) Distribution of patients according to endoscopy results ‘Zargar classification)

Organe	Lésion	Effectif	Pourcentage (%)
Œsophage	Stade I	1	25
	Stade IIa	2	50
	Stade IIIb	1	75
Estomac	Stade I		25
	Stade III		25
Duodénum	Absente	0	0
Fistule œso-trachéale	Absente	0	0

Sur le plan thérapeutique, tous les patients ont bénéficié d'antiémétiques afin d’éviter les efforts de vomissements. La douleur a été prise en charge par des antalgiques de palier 2 par voie intraveineuse (IV). Les inhibiteurs de la pompe à proton (IPP) ont été utilisés pour lutter contre les effets du reflux gastroœsophagien (RGO) chez tous les patients. Une antibioprophylaxie IV par céphalosporine de 3^e^ génération a été réalisée chez 12 patients (71 %). Une corticothérapie à base de prednisolone IV à la dose de 1 g/1,73 m^2^ par jour a été utilisée afin de limiter ou de prévenir les sténoses chez 9 patients (53 %). L'alimentation par voie parentérale a été utilisée chez 7 patients (41 %). La reprise de l'alimentation a été immédiate chez 5 patients (29 %). Des dilatations endoscopiques ont été réalisées chez 2 patients (12 %), après 4 semaines d’évolution avec succès.

**Figure 4:**
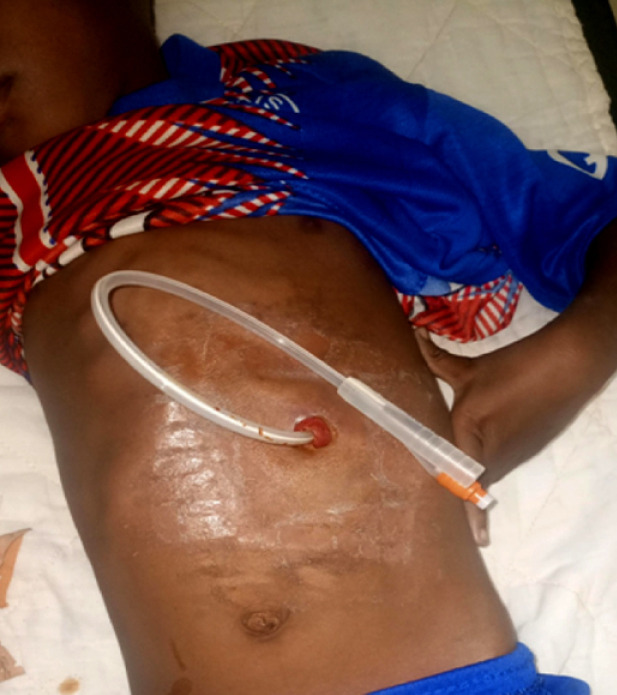
Gastrostomie d'alimentation transitoire en urgence chez un enfant Emergency transient feeding gastrostomy in a child

**Figure 5:**
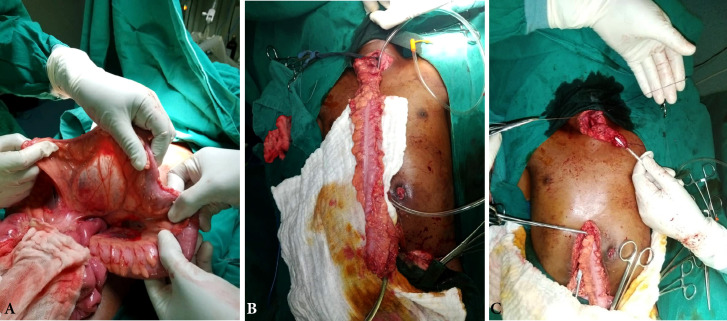
Procédure d'une œsophagoplastie colique : (A = préparation du transplant et choix du pédicule nourricier; B = mesure de la taille du transplant colique; C = mise en place du transplant colique) Colonic esophagoplasty procedure: (A = preparation of the transplant and choice of the feeder pedicle; B = measurement of the size of the colonic transplant; C = placement of the colonic transplant)

**Figure 6:**
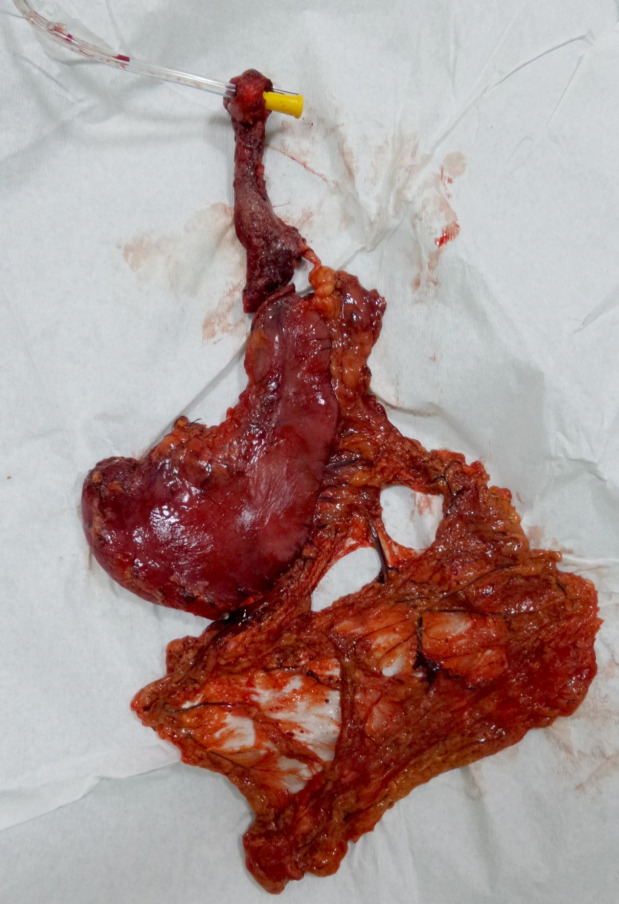
Pièce opératoire d'un stripping de l’œsophage associé à une gastrectomie totale Operative specimen of stripping of the esophagus associated with a total gastrectomy

Un traitement chirurgical en urgence a été effectué chez 7 patients (41 %) : 3 patients ont bénéficié de gastrostomie d'alimentation transitoire (Fig. 4); chez 3 autres il a été pratiqué des œsophagoplasties par transplant colique (Fig. 5) et 1 dernier a été traité par un stripping de l’œsophage associé à une gastrectomie totale (Fig. 6). L’évolution postopératoire a été marquée par une fistule de l'anastomose œsocolique chez un patient, pour lequel un traitement conservateur a été réalisé avec une bonne évolution. La durée moyenne de séjour hospitalier était de 5 jours (1 à 32 jours). Aucun décès n'a été répertorié dans notre série.

## Discussion

Cette étude est la première dans notre contexte à analyser les résultats de la prise en charge médico-chirurgicale en urgence des lésions œsophagiennes liées aux ingestions de produits caustiques chez l'enfant. En effet, il s'agissait d'une étude préliminaire incluant 17 patients admis dans un hôpital nouvellement construit. L’étude a permis de faire ressortir les circonstances de l'ingestion des produits caustiques qui était accidentelle. Les types de produits utilisés sont : la soude caustique (utilisée dans la fabrication artisanale de savon et comme déboucheur des canalisations), l'acide chlorhydrique (utilisé dans l'orpaillage artisanal), l'eau de Javel (pour la désinfection des aliments et le blanchissage du linge blanc) et le défrisant pour cheveux. L’étude a également permis de relever les conditions d'admission aux urgences ainsi que les résultats de la prise en charge montrant qu'une intervention chirurgicale était nécessaire chez 7 patients (41 %).

Notre hôpital dispose d'un personnel et d'un plateau technique adéquat permettant la prise en charge en urgence des cas d'ingestion de produit caustique. Les limites de cette étude sont d'une part son caractère rétrospectif ne permettant pas de collecter toutes les données et d'autre part le retard dans la consultation médicale, elle-même liée au manque de moyens financiers. Ce retard est à la base de la non-réalisation systématique d'une endoscopie digestive haute chez tous les patients. En effet, le degré de sévérité des lésions digestives initiales conditionnant la prise en charge est évalué par l'endoscopie grâce à l'utilisation de la classification de Zargar ou celle de Di-Costanzo [2]. Cette endoscopie doit être réalisée dans les 6 à 24 heures après l'ingestion. Les lésions peuvent être sous-évaluées si l'endoscopie est réalisée avant 6 heures, et il existe un risque de perforation iatrogène si elle est réalisée au-delà des 24 heures [9].

Dans notre étude, le nombre de cas d'ingestion de produit caustique chez l'enfant colligé en 2 ans ne reflète pas la réalité sur la prévalence des cas d'ingestion de caustique que pourrait enregistrer notre hôpital. En effet, ces enfants ne consultent pas systématiquement.

Seuls les cas sérieux sont généralement admis aux urgences. L'absence de mesures de prévention et de législation concernant les emballages fait que ces accidents surviennent de plus en plus fréquemment dans les pays en développement. En Sierra Leone, une étude réalisée dans un service de chirurgie pédiatrique avait permis de recenser 40 cas entre 2001 et 2005 [6]. Dans les pays du Maghreb, l'ingestion de caustique représente l'une des principales indications de la fibroscopie digestive en urgence chez les enfants [17]. En Turquie, ils représentaient 2,2 ***%*** de tous les patients du service de chirurgie infantile en 1997, 5,9 % en 2003 et 8,1 % en 2004 [1].

Dans notre étude, les manifestations cliniques sont très variées et intriquées. La clinique reste un indicateur peu suffisant pour évaluer la sévérité des lésions. Une endoscopie haute est nécessaire. La présence des lésions endoscopiques de stade IIIb est une indication pour la réalisation d'un scanner thoraco-abdominal injecté afin d’évaluer le degré de l'extension de la nécrose transpariétale œsophagienne; avec une sensibilité de 81,2 %, et une spécificité de 80,8 % [8].

La prise en charge initiale n'est pas consensuelle, cependant, il est admis qu'elle doit se faire en milieu spécialisé et de façon pluridisciplinaire [14]. Le traitement médical vise à empêcher l'inhalation et les vomissements par les antiémétiques et à lutter contre les effets du reflux gastro-œsophagien par les IPP [19]. Bien que nous ayons utilisé une antibioprophylaxie chez la majorité des patients (71 %) dans le but de prévenir les sténoses en cas de lésions sévères. À ce jour, il n'existe pas de preuves scientifiques claires, sur l'utilité des antibiotiques dans ce domaine [7]. La corticothérapie à forte dose par voie intraveineuse pendant trois semaines a été utilisée chez 53 % des patients. Cependant, les auteurs d'une méta-analyse publiée en 2018 n'ont trouvé aucune preuve appuyant l'utilisation de corticostéroïdes pour prévenir les sténoses œsophagiennes suite à l'ingestion 

de produits caustiques [11]. La jéjunostomie d'alimentation ou l'alimentation parentérale sont indiquées chez les patients avec des lésions de stade III [4]. Les lésions de nécrose diffuse et circonférentielle (stade IIIb) constituent une indication d’œsophagectomie par stripping. Une gastrectomie totale peut être associée à l’œsophagectomie si nécessaire. Les plasties œsophagiennes par transplant colique, gastrique ou iléal, avec ou sans résection de l’œsophage cicatriciel, s'adressent aux sténoses étendues ou multiples; elles sont habituellement résistantes aux traitements endoscopiques [12]. La réalisation de ces plasties œsophagiennes, dans le contexte des pays en développement, nécessite souvent une évacuation vers des centres médicaux plus équipés. C'est ainsi qu'une étude réalisée dans le service de chirurgie pédiatrique du CHU vaudois (Lausanne, Suisse), sur une période de 35 ans, par Olivier Reinberg, révèle que les enfants de pays de l'Afrique de l'Ouest constituent la majorité des enfants qui ont bénéficié de remplacement œsophagien pour ingestion de caustique. Ils étaient 251 sur les 282 remplacements œsophagiens réalisés dans leur service [16].

Le risque de développer un carcinome épidermoïde sur l’œsophage cicatriciel qui est resté dans le circuit alimentaire est estimé à 1000 fois supérieur à celui de la population générale, d'où la nécessité d'une surveillance à long terme. Un intervalle moyen de 40 ans a été rapporté entre l'exposition aux caustiques et son développement [15].

## Conclusion

L'ingestion accidentelle de produits caustiques peut occasionner des lésions œsophagiennes engageant dans l'immédiat le pronostic vital et, à plus long terme, fait courir le risque de lourdes séquelles. Dans notre étude, les lésions œsophagiennes chirurgicales sont présentes chez plus du tiers des patients. L'endoscopie digestive haute et le scanner injecté ont permis d’évaluer la sévérité des lésions. La prise en charge des formes sévères est difficile et se fait de manière pluridisciplinaire. La prévention reste le meilleur traitement.

## Contribution des auteurs

Les auteurs ont contribué à la conception du travail; à l'acquisition, l'analyse ou l'interprétation des données; et à la rédaction ou la révision critique du manuscrit comme suit : Rabiou SANI 30 %, Aliou ZABEIROU 40 %, Illé SALHA, Ibrahim ISSOUFOU ALZOUMA, Boubé DJAFAROU ABARCHI, Lassey JAMES DIDIER, Rachid SANI et Habibou ABARCHI 5 % chacun. La version à publier a été approuvée par tous les auteurs, et ils ont accepté d’être responsables de tous les aspects du travail.

## Conflits d'intérêts

Les auteurs ne déclarent aucun lien d'intérêt.
